# Identification of novel variants in *MYO15A*, *OTOF*, and *RDX* with hearing loss by next‐generation sequencing

**DOI:** 10.1002/mgg3.808

**Published:** 2019-06-28

**Authors:** Xuejing Bai, Shiyan Nian, Lei Feng, Qingrong Ruan, Xuan Luo, Mengna Wu, Zefeng Yan

**Affiliations:** ^1^ Department of Laboratory The Sixth Affiliated Hospital of Kunming Medical University Yuxi P.R. China; ^2^ Department of Laboratory People’s Hospital of Yuxi City Yuxi P.R. China

**Keywords:** *MYO15A*, next‐generation sequencing, nonsyndromic hearing loss, *OTOF*, *RDX*

## Abstract

**Background:**

Nonsyndromic hearing loss (NSHL) is the most common sensorineural disorder and one of the most common human defects. Autosomal recessive inheritance accounts for a huge percentage of familial cases. Next‐generation sequencing (NGS) is a powerful molecular diagnostic strategy for NSHL. The combination of a microarray gene chip and NGS can better delineate the etiology and genetic cause of deafness in many cases.

**Methods:**

One hundred and thirty‐one unrelated students with NSHL who attend a special education school in Yunnan Province were recruited. Firstly, four common deafness‐related genes (*GJB2*, *GJB3*, *SLC26A4*, and mtDNA *12S rRNA*) were evaluated for mutations using a microarray kit. Furthermore, 227 known human deafness genes were sequenced to identify the responsible genetic variant of the proband in three Chinese families with autosomal recessive hearing loss. The mutational status of family members of the probands was validated by Sanger sequencing.

**Results:**

Five novel variants were found in three families using NGS. In family 1, we identified compound heterozygosity at the *MYO15A* (OMIM, #600316), including an duplication variant c.3866dupC, p.His1290Alafs*25 and a 3‐bp deletion (c.10251_10253del, p.Phe3420del), resulting in protein length changes and premature protein truncation, respectively. In family 2, two affected siblings from a consanguineous Chinese Dai family harbored an c.1274G>C, p.Arg425Pro missense variant in the *OTOF* (OMIM, #601071). In family 3, we identified compound heterozygosity for c.129_130del, p.His43Glnfs*28 and c.76_79del, p.Lys26* in the *RDX* gene (OMIM, #611022).

**Conclusion:**

Five novel variants were found in three families with NSHL. Our findings extend the mutational spectrum in deafness‐related genes and will help physicians in better understanding the etiology of hearing loss.

## 1 INTRODUCTION

Hearing loss (HL) is a sensory defect that affects 1–3 in every 1,000 newborns worldwide, and half of these cases are attributed to genetic factors (Shen et al., 2017). HL can be classified according to age of onset, signs and symptoms, severity, and genetic basis. Based on pure tone audiometry, HL is categorized as conductive, sensorineural, and mixed. Prelingual or postlingual are used to distinguish the time of onset. HL is divided into syndromic and nonsyndromic by its accompanying signs and symptoms, and classified into four grades (mild, moderate, severe, and profound) according to severity (Li & Ping, 2002). Nonsyndromic hearing loss (NSHL) makes up a large proportion of HL. NSHL shows different modes of inheritance, including autosomal recessive (AR), autosomal dominant (AD), X‐linked, and mitochondrial (Taghipour‐Sheshdeh et al., 2018). AR transmission accounts for 75%–85% of all cases, while AD inheritance accounts for 15%–25% of cases. A small proportion of cases (1%–2%) show X‐linked or mitochondrial inheritance (Atik, Bademci, Diaz‐Horta, Blanton, & Tekin, 2015).

Previous studies have described variants in several genes that are closely related to NSHL in China, such as mtDNA*12S rRNA*, *SLC26A4*, *GJB2,* and *GJB3* (Lu et al., 2011). A microarray kit with these four genes is used for routine screening. However, HL is an extremely heterogeneous disease, which makes molecular diagnosis challenging. Next‐generation sequencing (NGS) is a strategy that can overcome this problem and play an important role in prognosis evaluation, clinical management, and prenatal diagnosis. So far, more than 60 genes have been identified and at least 1,949 pathogenic variants have been reported for NSHL (http://hereditaryhearingloss.org/).

Here, we used a microarray kit for the initial screening of four genes, and then NGS was applied to explore causal variants in cases for which no variants were detected using the microarray. Five new variants were found in *MYO15A*, *OTOF,* and *RDX*.

## 2 MATERIALS AND METHODS

### 2.1 Subjects

The study protocol was approved by the Review Board of the People's Hospital of Yuxi City (Yunnan, China). One hundred and thirty‐one unrelated students with NSHL were recruited who studied at a special education school in Yunnan Province, China. Informed consent was obtained from all participants or their guardians. All participants underwent a standard test, including clinical and audiometric assessments to exclude possible nongenetic factors of HL. Pure tone average (PTA) was used for the clinical hearing assessment. According to the WHO‐1997 hearing impairment classification standard, the pure tone thresholds at 500 Hz, 1000 Hz, 2,000 Hz, and 4,000 Hz were determined, and the mean value was calculated. Peripheral blood samples were collected in ethylenediamine tetraacetic acid‐K2 anticoagulant tubes in accordance with the standard procedure. We finally selected three families with a family history for this study. Family 2 is a Chinese Dai family and four members of the family participated in this study. The other two families are both Han. Bilateral prelingual deafness was observed in the proband and their siblings of each family, but their parents had normal hearing (Figure 1a).

**Figure 1a:**
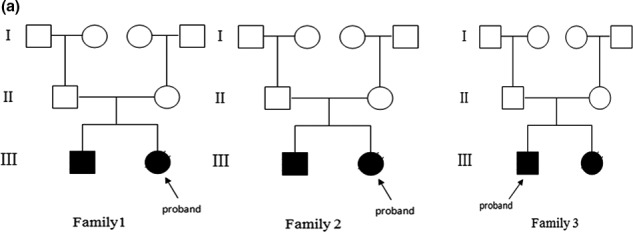
The family pedigree. The arrow represents the proband, black represents deafness, and the cross represents death

### 2.2 Variant detection and variant analysis

All 131 individuals enrolled in our study were subjected to a preliminary screening using a microarray method with the Hereditary Deafness Gene Mutation Detection Kit (CapitalBio Technology, Beijing, China). The kit was designed to detect nine well‐documented variants in four deafness associated genes, including *GJB2*: c.35delG, c.176_ 191del6, c.235delC, and c.299delA; *GJB3*:c.538C>T; *SLC26A4*:c.2168A>G and c.*IVS7*‐2A>G; and *12S rRNA*: m.1494C>T and m.1555A>G. In addition, NGS was applied following the standard protocol by CapitalBio Technology. Genomic DNA was extracted from peripheral blood samples of all subjects. At least 0.5 μg of genomic DNA from the probands were sheared by Covaris S2 (Covaris, MA, USA). AMPure beads (Beckman Coulter, CA, USA) were used for purification recycling, followed by enrichment and PCR, and then Ion PI Chip v3 and a BSE4000 sequencing machine were used for high‐throughput sequencing. Finally, the sequences were compared to the human reference genome (hg19, NCBI release GRCh37) using the TMAP Alignment Program, and variants were analyzed using Torrent Variant Caller (Xia et al., 2015).

## 3 RESULTS

### 3.1 Clinical findings

In this study, in the preliminary hereditary deafness gene variant screening using the microarray, 27 out of 131 individuals (28.3%) were found with common, previously reported variants. However, using NGS, we detected five new variants in three families. In these three families, bilateral severe or profound sensorineural hearing loss with thresholds over 70 dBHL was revealed by PTA in three probands. No inner ear abnormalities were discovered by magnetic resonance imaging in the proband in each family (Figure 1bb). All parents have normal hearing, while the probands’ siblings also have abnormal hearing.

**Figure 1b:**
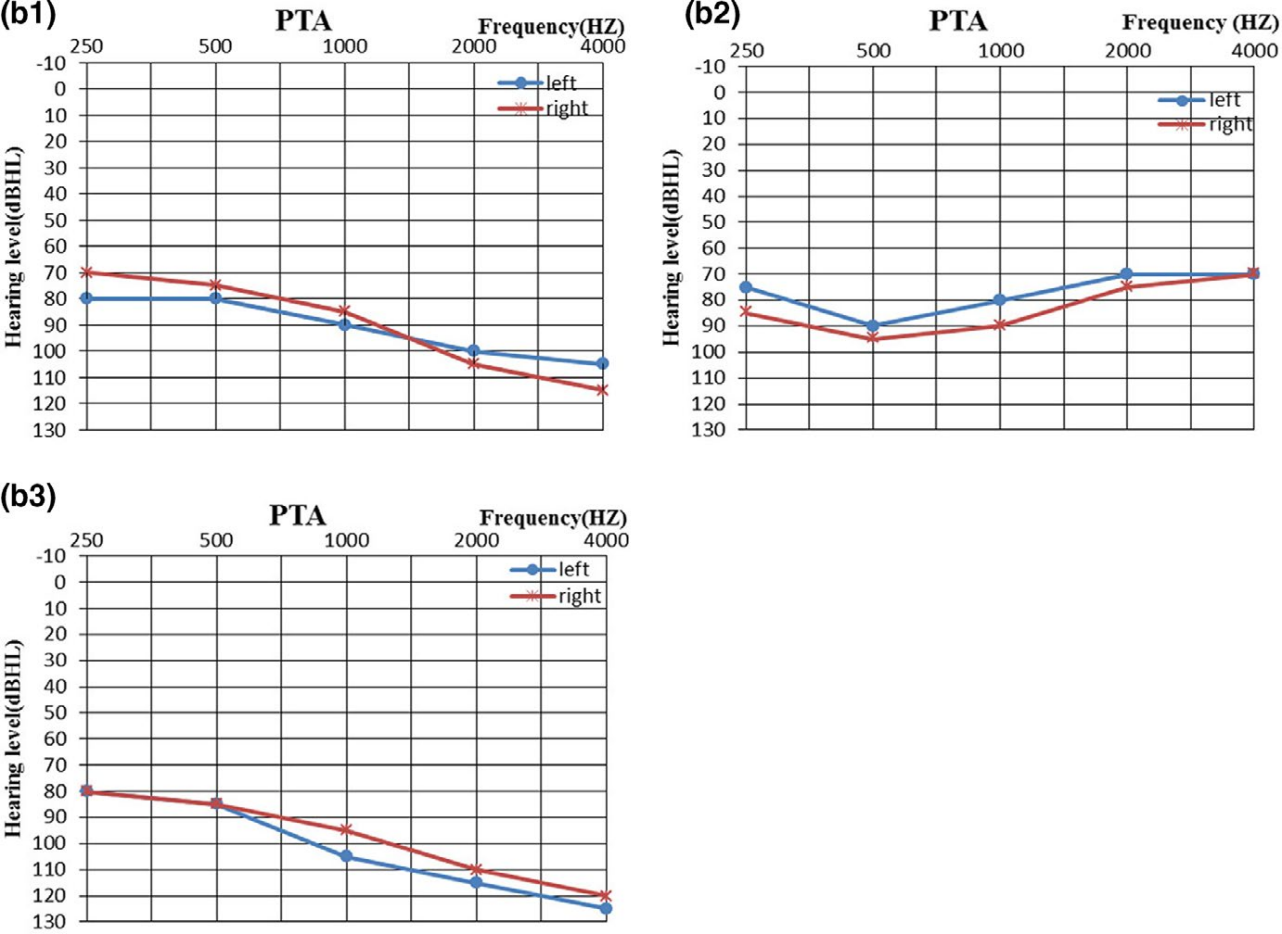
(1b‐1, 1b‐2, 1b‐3). The PTA results of three probands. The average thresholds in the left and right ears of proband 1 were 93.75 dBHL and 95 dBHL, respectively; 77.5 dBHL and 82.5 dBHL, respectively for proband 2; and 107.5 dBHL and 102.5 dBHL, respectively, for proband 3. PTA, pure tone average

### 3.2 NGS and variant analysis

The American College of Medical Genetics and Genomics (ACMG) guidelines recommend that specific standard terminology is used to describe variants identified in genes that cause Mendelian disorders, including pathogenic, likely pathogenic, uncertain significance, likely benign and benign variants (Richards et al., 2015). The variants detected in the three families are shown in Table 1. In family 1, we identified compound heterozygosity in the *MYO15A* gene, including c.3866dupC, p.His1290Alafs*25 and c.10251_10253del, p.Phe3420del, both of which are very rare variants. The c.3862dupC, p.His1290Alafs*25 variant is likely pathogenic as it results in premature protein truncation. The c.10251_10253del, p.Phe3420del variant has uncertain significance, and this variant can lead to changes in protein length, which make it unable. This very rare variant has not been reported before, is located at an amino acid position highly conserved throughout evolution from mammals to birds (Figure 2a) and is not listed in the Genome Aggregation Database (gnomAD) or the Exome Aggregation Consortium (ExAC) database.

**Table 1:** The variants detected in the three families

	Gene	Transcript	Chromosome location (GRCh37/hg19)	Nucleotide changes	Amino acid change	Sequencing analysis			
						Proband	Sibling	Father	Mother
Family 1	*MYO15A*	NM_016239	chr17:18029765 chr17:18075505	c.3862dupC c.10251_10253del	p.His1290Alafs*25 p.Phe3420del	Heterozygous Heterozygous	Heterozygous Heterozygous	Heterozygous	Heterozygous
Family 2	*OTOF*	NM_001287489	chr2:26706448	c.1274G>C	p.Arg425Pro	Homozygous	Homozygous	Heterozygous	Heterozygous
Family 3	*RDX*	NM_001260494	chr11:110128844 chr11:110128895	c.129_130del c.76_79del	p.His43Glnfs*28 p.Lys26*	Heterozygous Heterozygous	Heterozygous Heterozygous	Heterozygous	Heterozygous

**Figure 2a:**
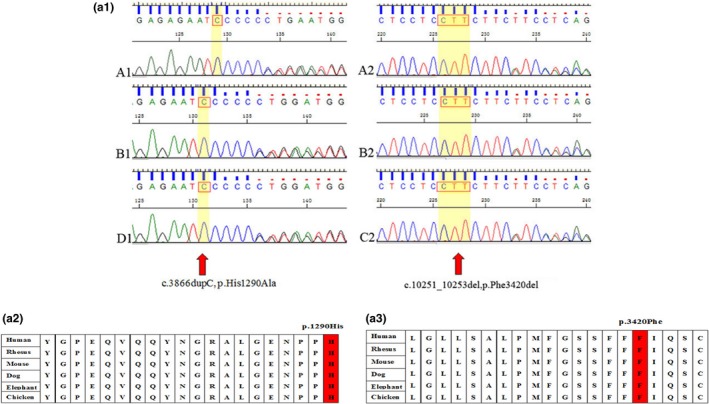
(2a‐1, 2a‐2, 2a‐3). Family 1, cross‐species multiple alignments of the Myosin 15a polypeptide. The amino acids at positions p.1290His and p.3420Phe are highly conserved from human to chicken

In family 2, two affected siblings from a consanguineous Chinese Dai family with AR deafness 9 (DFNB9) were identified as harboring a p.Arg425Pro missense variant in the *OTOF* gene caused by a homozygous 1274C‐G transition, and the heterozygous variant was identified in both of their unaffected parents. This missense variant is likely to be a pathogenic variant by the ACMG guidelines. This was not listed in the gnomAD or ExAC databases, and has not been reported before. The conserved sequence is shown in Figure 2b.

**Figure 2b:**
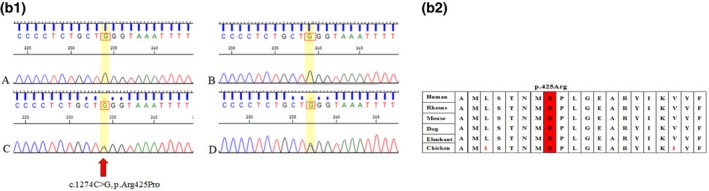
(2b‐1, 2b‐2). Cross‐species multiple alignments of oterfrin. The amino acid at position p.425Arg is highly conserved from human to chicken

In family 3, the proband was identified as having compound heterozygosity for a 2‐bp deletion (c.129_130del) and a 4‐bp deletion (c.76_79del) in the *RDX* gene. Both are likely to be pathogenic variants. They are predicted to cause premature stop codons that influence the entire second FERM domain until the N terminus. The FERM domain is a conserved protein module found in a number of proteins that can mediate protein–protein interactions. Subsequently, compound heterozygosity was detected in the proband's sister for c.129_130deland c.76_79del in the *RDX* gene, and his parents were verified as carrying c.129_130del and c.76_79del, respectively, by Sanger sequencing. These are both very rare variations that have not been reported before, are highly conserved (Figure 2c) and are not listed in the gnomAD or ExAC databases.

**Figure 2c:**
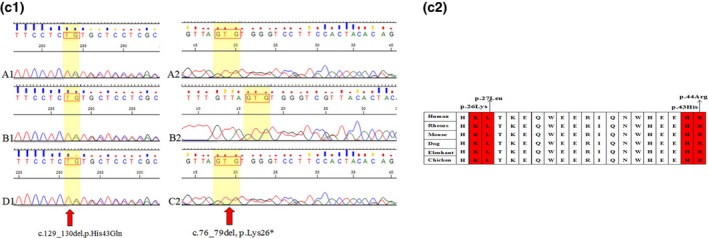
(2c‐2, 2c‐2). Cross‐species multiple alignments of radixin. The amino acids at positions p.26Lys and p.43His are highly conserved from human to chicken. Figure 2. Appendix: The sequencing peak of each proband and their parents. A. The proband; B. The siblings of the proband; C. The father of the proband; and D. The mother of the proband

## 4 DISCUSSION

In our study, we found five novel variants in three genes. Among them, *MYO15A* encodes myosin, *OTOF* encodes otoferlin, and *RDX* encodes radixin. When these genes are mutated, they may cause changes in protein length or function.

Myosins (MYO) are actin proteins that produce a powerful force for cytoskeletal movement and assist in the production of ATP. Based on their variable C‐terminal binding domain, they are classified as conventional MYO (class II) and unconventional MYO (class I and III‐XV). The proteins encoded by these genes play an important role in human hearing. Variants in three different genes result in the loss of function of the coding protein and progressive NSHL. The expression of these genes is highly restricted and is strongest in the retina and cochlea. Members of the myosin superfamily (*MYO7A*, *MYO15A*, and *MYO6*) are involved in voice conduction, but variants in *MYO15A* are now considered to be one of the most common causes of nonsyndromic autosomal recessive hearing loss (ARNSHL) (Belyantseva et al., 2005).

The variant in the *MYO15A* is now a globally noted cause of ARNSHL and this deafness locus has been designated DFNB3 (OMIM, #600316). By comparing different ethnic groups, the *MYO15A* is considered to be the third most common deafness gene, and all domains and motifs of protein coding sequence by *MYO15A* gene have been identified. *MYO15A* is located on chromosome 17p11 and has a length of 71kb, and the myosin XVA polypeptide chain is encoded by 66 exons, consisting of 3,530 amino acids and is 395 kDa in size (Rehman et al., 2016). It includes a long N‐terminal extension encoded by exon 2, an N‐terminal motor domain, two light chain binding Isoleucine—glutamine (IQ) motifs, and a tail region containing two myosin‐tail homology 4 domains, two band 4.1/ezrin/radixin/moesin (FERM) domains, an Src‐homology‐3 domain and a C‐terminal class I PDZ‐ligand domain (Figure 3a). Wang et al. initially revealed two missense mutations and one nonsense mutation affected individuals from three unrelated *DFNB3* families from Bangladesh and India (Wang et al., 1998). Another variant was subsequently found (c.6442T>A) (Reiisi, Tabatabaiefar, Sanati, & Chaleshtori, 2016), and there are now 198 variants in *MYO15A* that have been reported and reviewed in Venezia, Martin, Hickok, & Richards, (2019).

**Figure 3a:**
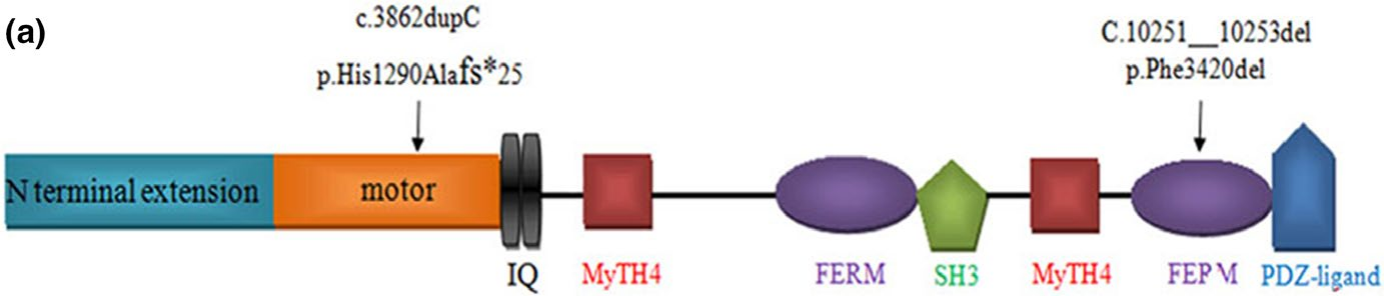
The schematic structure and mutations of the human *MYO15A* gene. The black arrows indicate the positions of the variants

In this study, an duplication variant (c.3866dupC, p.His1290Ala) and a 3‐bp deletion (c,10251_10253delCTT, p.Phe3420del) were detected. p.His1290Ala is a likely pathogenic variant, and it can lead to early termination of its coding peptide chain, the formation of truncated proteins or degradation. p.Phe3420del is judged as having uncertain significance, but this variant may prevent the normal functioning of its original protein. The three‐dimensional structure of the protein is shown in Figures 3b and 3c.

**Figure 3b:**
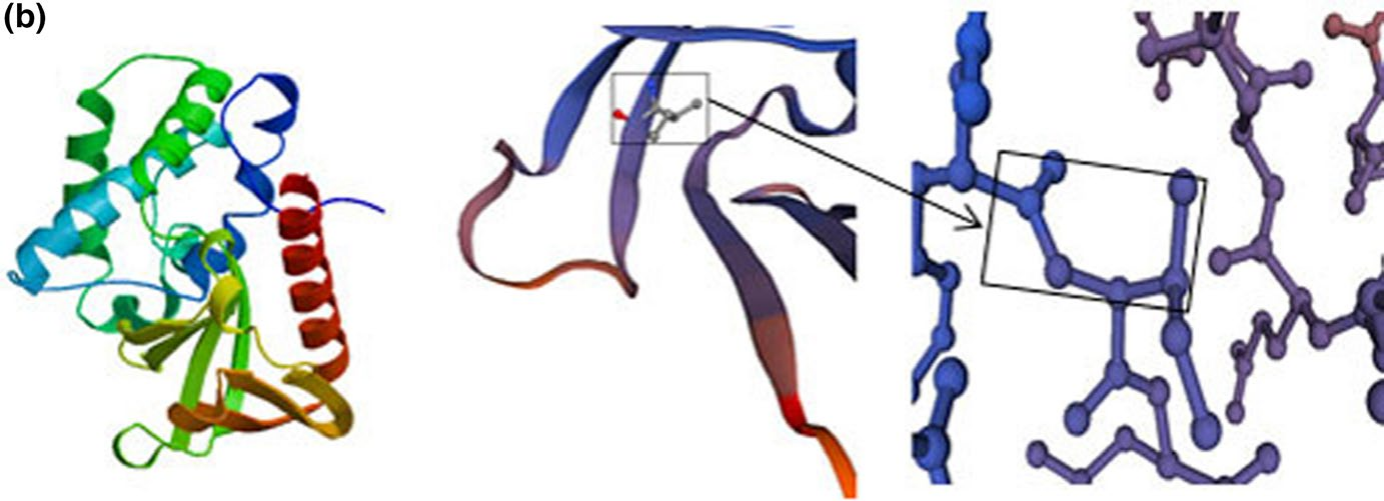
Structure of mutant *MYO15A*. A. p.Phe 3420del and the 3420 change into isoleucine

**Figure 3c:**
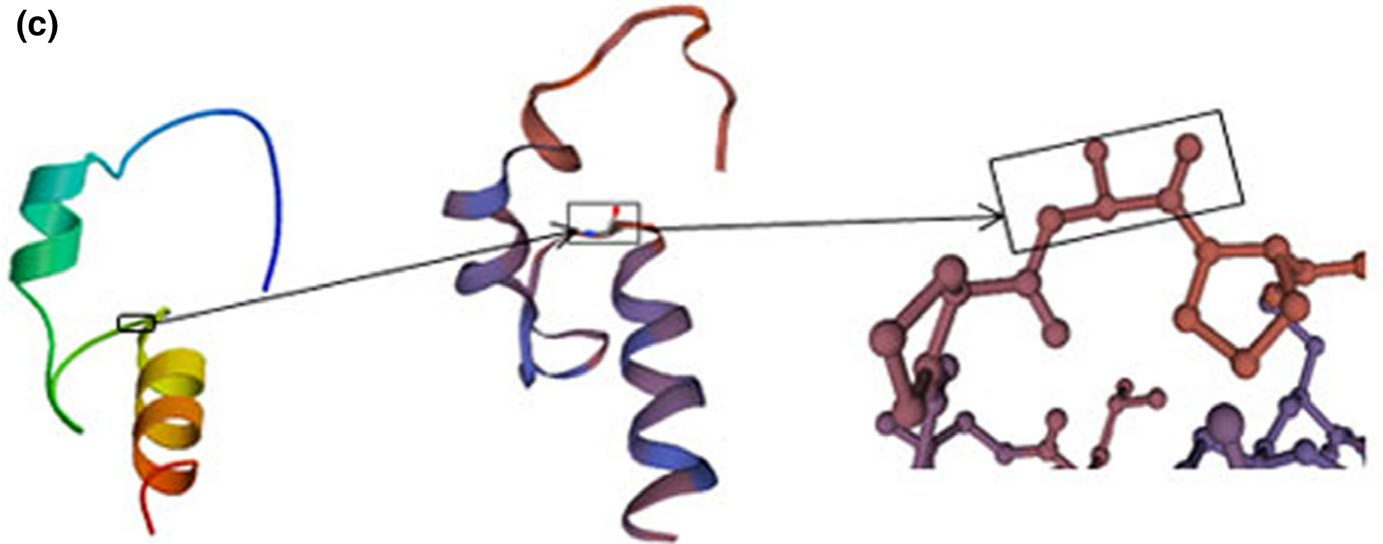
Structure of mutant *MYO15A*. p.His1290 Alafs*25, which leads to the formation of a truncated protein or degradation

*MYO7A* is located on 11q13.5 and consists of 55 exons, encoding a myosin composed of 2,215 amino acids with a short tail. Variants in this gene cause DFNB2 (OMIM, #600060) and DFNA11 (OMIM, #601317) forms of deafness. More than 340 different variants in myosin VIIA have been reported to be related to congenital syndrome deafness with retinitis pigmentosa (Usher syndrome) type 1 and type 2. Weil et al. showed that the efficiency of splicing can decrease when the last nucleotide of exon 15 contains a G to A transition (Weil et al., 1997). Subsequently, Chenget et al. found a patient with compound heterozygous variants, c.5168+1G>A and c.6070C>T, detected by high‐throughput sequencing (Cheng et al., 2018). Razmara et al. discovered other compound heterozygous variants (c.1708C>T and c.3751G>C) in an Iranian family. This c.3751G>C variant is located in the MYTH4 protein domain which is a pivotal domain for the myosin function, and its variant is associated with NSHL (Razmara, Bitarafan, Esmaeilzadeh‐Gharehdaghi, Almadani, & Garshasbi, 2018). Li et al. found a pathogenic variant (c.2011G>A) in a Chinese family in addition to genetic cosegregation (L. Li et al., 2018). In addition, four of the nine variants detected in the past 10 years were in Chinese families, and these variants are not uncommon in China.

Melchionda et al. found the *MYO6* gene (DFNA22) located on 6q13 in a family and identified a missense variant in 2002 (Melchionda et al., 2001). Ahmed et al. found that the deafness locus was designated DFNB37 in three Pakistani families, which is another genetic pattern of *MYO6*, and found a frameshift variant (36‐37inst), an uncertain significance variant (p.Arg1166X) and a missense variant (p.Glu216Val) through sequencing analysis(Ahmed et al., 2003).

The *OTOF* gene encodes otoferlin and is an autosomal recessively inherited genetic locus (DFNB9), which is located in 2p23.3. *OTOF* variants are known to lead to ARNSHL. Otoferlin has two isoforms, of which the short isoform has three C2 domains and one carboxylate transmembrane domain. The long isoform consists of 1997 amino acids (aa), including six C2 domains: C2A (2–97 aa), C2B (255–353 aa), C2C (418–529 aa), C2D (961–1068aa), C2E (1,493–1592 aa), and C2F (1733–1863 aa) (Figure 4a‐1). Otoferlin is homologous to the *Caenorhabditis elegans* spermatogenesis factor FER‐1 and human dysferlin (Motavaf, Soveizi, Maleki, & Mahdieh, 2017). Roux et al found that otoferlin may be involved in vesicular membrane fusion and is expressed in hair cells, which correlates with afferent synaptogenesis. Otoferlin localizes within ribbon‐associated synaptic vesicles, and the function of otoferlin may be related to endocytosis (Roux et al., 2006). Variants in *OTOF* have been reported as the main cause of nonsyndromic recessive auditory neuropathy spectrum disorder (Tang et al., 2017). Until 2017, at least 93 variants have been summarized (Meena & Ayub, 2017). To date, 21 additional *OTOF* variants have been identified. In this study, we detected a c.1274G>C missense variant. This variant was located in an important part of this protein. The amino acid sequences of different species are highly conserved, and according to the ACMG guidelines, it is likely to be a pathogenic variant.

**Figure 4a‐1:**
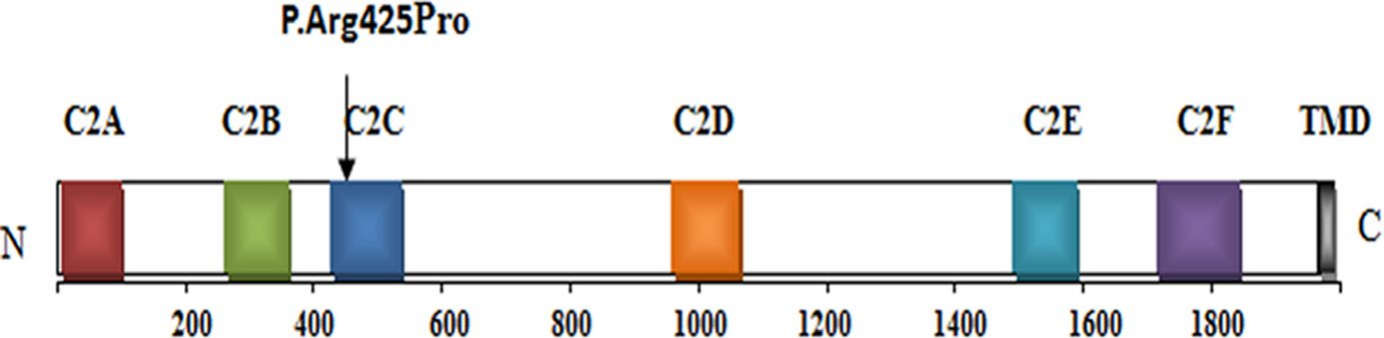
The schematic structure and mutations of the human *OTOF* gene. The black arrow indicates the positions of the variants. TMD: the transmembrane domain

The *RDX* gene is located at 11q23 as detected by fluorescence in situ hybridization, and is an autosomal recessive genetic locus (DFNB24). A truncated version representing the pseudogene (*RDXP2*) is located at Xp21.3. From the N terminus to the C terminus is a FERM domain, a helical a‐domain and a carboxyl terminal domain (Figures 4a‐1 and 4a‐2) (Wilgenbus, Milatovich, Francke, & Furthmayr, 1993). Another pseudogene (*RDXP1*), appearing to lack an intron and located at 11p, was detected by Southern blot and PCR analysis. A variety of different splicing transcriptional variants have been identified, encoding different subtypes. Wilgenbus et al. cloned and sequenced the human radixin cDNA and found that the protein is highly homologous to ezrin and moesin (Wilgenbus et al., 1993). Multiple tissues and organs can produce radixin, mainly the adrenal glands. Pataky et al. demonstrated that radixin is expressed at the base of hair bundles in chicken, frog, mouse and zebrafish, and they concluded that radixin may participate in anchoring the “pointed” ends of actin filaments to the membrane in stereocilia (Pataky, Pironkova, & Hudspeth, 2004). There are a few reports of *RDX* variants associated with deafness in existing research. Khan et al. found two variants in *RDX*, 1732G>A, p. D578N and c.1404_1405 insG p.A469fsX487 associated with sensorineural hearing impairment in two consanguineous families and a c.463C>T transition substitution (Khan et al., 2007). In 2009, Shearer et al. found a new homozygous splice site variant (c.698+1G>A) in an Iranian family (Cabanillas et al., 2018). The c.76_79del, p.Lys26* and c.129_130del, p.His43Glnfs*28 frameshift variants were detected in our study, which can prevent the translation of the encoded peptide chain and form truncated proteins or lead to decomposition. The three‐dimensional structure of the protein is shown in Figure 4b. According to the ACMG guidelines, they are both considered to be pathogenic variants.

**Figure 4a‐2:**
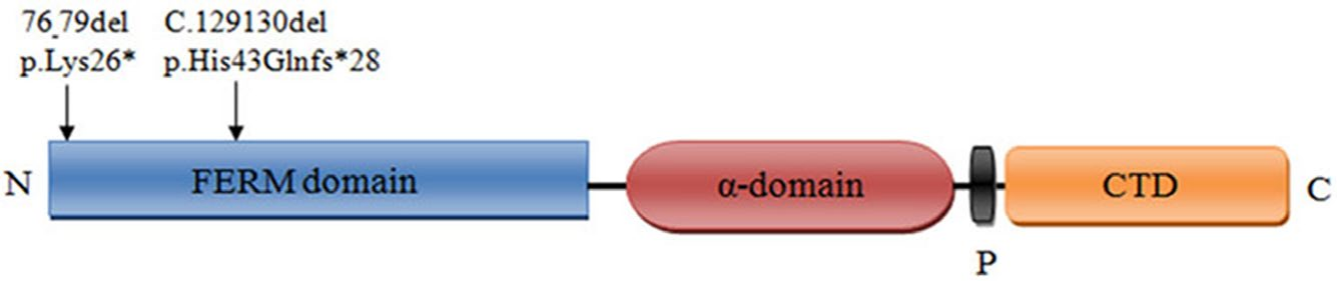
The schematic structure and mutations of the human *RDX* gene. The black arrows indicate the positions of the variants

**Figure 4b:**
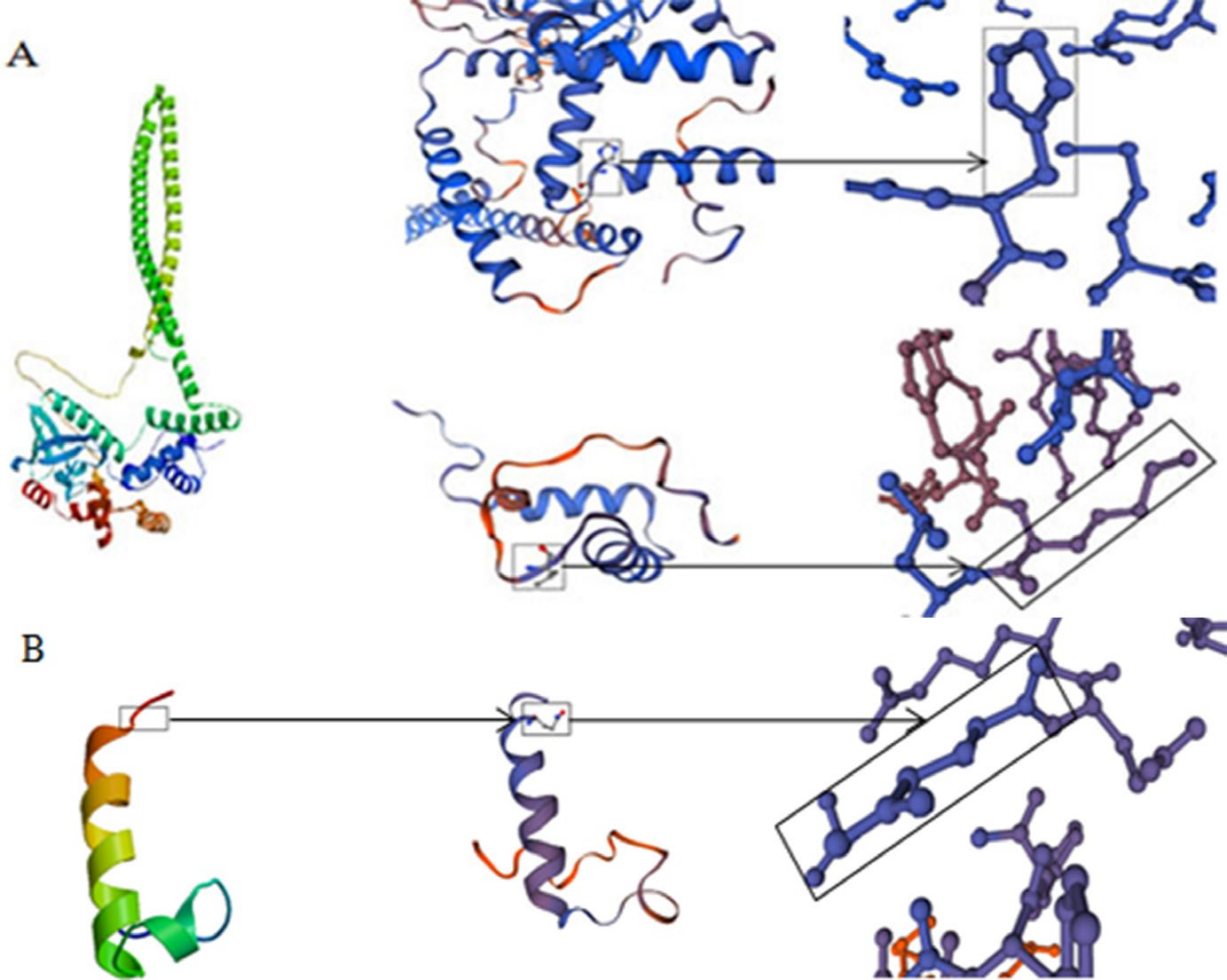
Structure of wild‐type and variant Radixin. A. 26th and 43rd amino acids of the wildtype Radixin. B. p.His43Glnfs*28, which leads to the formation of a truncated protein or degradation

In conclusion, five novel variants expand the mutational spectrum of *MYO15A, OTOF* and *RDX* in the Chinese population, which will contribute to the clinical understanding of HL caused by variants in these genes and will help physicians in better understanding the etiology of HL.

## CONFLICT OF INTERESTS

The authors declare that they have no competing interests.

## AUTHOR CONTRIBUTIONS

Xuejing Bai drafted the manuscript, analyzed and interpreted the data, revised the manuscript, approved the final version, and agreed to be accountable for all aspects of the work; Shiyan Nian designed the work partially, interpreted the data, revised the manuscript critically, approved the final version, and agreed to be accountable for all aspects of the work; Lei Feng planned the study, interpreted the data, revised the manuscript critically, approved the final version, and agreed to be accountable for all aspects of the work; Qingrong Ruan designed the work partially, interpreted the data, revised the manuscript critically, approved the final version, and agreed to be accountable for all aspects of the work; Xuan Luo designed the work partially, interpreted the data, revised the manuscript critically, approved the final version, and agreed to be accountable for all aspects of the work; Mengna Wu designed the work partially, interpreted the data, revised the manuscript critically, approved the final version, and agreed to be accountable for all aspects of the work; Zefeng Yan designed the work partially, interpreted the data, revised the manuscript critically, approved the final version, and agreed to be accountable for all aspects of the work.

## ETHICS APPROVAL AND CONSENT TO PARTICIPATE

The study was approved by the Review Board of the People's Hospital of Yuxi City (Yunnan, China). All subjects recruited in this study provided written informed consent.

## CONSENT FOR PUBLICATION

The consents for publication of the individual person's data have been obtained from the person themselves or their parents, in the case of children.

## Data Availability

The dataset supporting the conclusions of this article is included within the article.
